# Sociodemographic characteristics of community eye screening participants: protocol for cross-sectional equity analyses in Botswana, India, Kenya, and Nepal

**DOI:** 10.12688/wellcomeopenres.17768.2

**Published:** 2023-11-16

**Authors:** Luke N Allen, Oathokwa Nkomazana, Sailesh Kumar Mishra, Bakgaki Ratshaa, Ari Ho-Foster, Hillary Rono, Abhiskek Roshan, David Macleod, Min Kim, Ana Patricia Marques, Nigel M Bolster, Matthew J Burton, Michael Gichangi, Sarah Karanja, Andrew Bastawrous

**Affiliations:** 1 Department of Clinical Research, London School of Hygiene & Tropical Medicine, London, UK; 2 Faculty of Medicine, University of Botswana, Gaborone, Botswana; 3 Nepal Netra Jyoti Sangh, Kathmandu, Nepal; 4 Peek Vision, Berkhamsted, UK; 5 Kitale County and Referral Hospital, Kitale, Kenya; 6 Sagarmatha Choudhary Eye Hospital, Lahan, Nepal; 7 International Centre for Eye Health, London School of Hygiene & Tropical Medicine, London, UK; 8 Ministry of Health, Nairobi, Kenya; 9 Kenya Medical Research Institute, Nairobi, Kenya

**Keywords:** sociodemographic, socioeconomic status, socioeconomic position, data collection, pragmatic research, embedded research, equity, global health, epidemiology, eye health, screening

## Abstract

**Background:**

Attendance rates for eye clinics are low across low- and middle-income countries (LMICs) and exhibit marked sociodemographic inequalities. We aimed to quantify the association between a range of sociodemographic domains and attendance rates from vision screening in programmes launching in Botswana, India, Kenya and Nepal.

**Methods:**

We performed a literature review of international guidance on sociodemographic data collection. Once we had identified 13 core candidate domains (age, gender, place of residence, language, ethnicity/tribe/caste, religion, marital status, parent/guardian status, place of birth, education, occupation, income, wealth) we held workshops with researchers, academics, programme implementers, and programme designers in each country to tailor the domains and response options to the national context, basing our survey development on the USAID Demographic and Health Survey model questionnaire and the RAAB7 eye health survey methodology. The draft surveys were reviewed by health economists and piloted with laypeople before being finalised, translated, and back-translated for use in Botswana, Kenya, India, and Nepal. These surveys will be used to assess the distribution of eye disease among different sociodemographic groups, and to track attendance rates between groups in four major eye screening programmes. We gather data from 3,850 people in each country and use logistic regression to identify the groups that experience the worst access to community-based eye care services in each setting. We will use a secure, password protected android-based app to gather sociodemographic information. These data will be stored using state-of-the art security measures, complying with each country’s data management legislation and UK law.

**Discussion:**

This low-risk, embedded, pragmatic, observational data collection will enable eye screening programme managers to accurately identify which sociodemographic groups are facing the highest systematic barriers to accessing care at any point in time. This information will be used to inform the development of service improvements to improve equity.

## Introduction

### Inequalities in eye health

Universal Health Coverage ^1^ and the principle of proportionate universalism ^2^ are responses to the fact that health outcomes are inequitably distributed across and between populations ^2– 4^. The inverse care law states that the supply of medical care is inversely proportional to need ^5^, and the most disadvantaged groups in society are often the least likely to attain good health outcomes ^6^.

Over one billion people currently live with visual impairment, levying major economic, social, and human costs ^7^. Eye conditions exhibit marker inter- and intra-national inequalities in disease rates, access to care, and outcomes, with poorer, rural women often facing the highest barriers to accessing care ^7– 9^.

In recognition of the enormous costs of preventable visual impairment, governments, health organisations and funding agencies are increasingly investing in national eye screening programmes ^10^. Embedding sociodemographic data collection into these programmes could help to illuminate the distribution of risks, disease burden, access, and service utilisation. These data can be used to identify the groups facing systematic barriers to care, and to inform targeted work to redress inequalities.

A large number of countries and implementing partners use screening programmes designed by the social enterprise Peek Vision. These programmes run on a suite of Peek apps that are used for data entry, screening, referral, and clinic check-in; albeit with local modifications to the organisational flow and structure of the screening programmes depending on population and context. Peek-based screening programmes are currently running in eleven low-and middle-income countries (LMICs), and four new programmes setting up in Botswana, India, Kenya and Nepal will screen hundreds of thousands of people this year ( Table 1). These four countries were chosen for the current project because they all have active Peek programmes led by institutions with active research interest and existing relationships with LSHTM.

**Table 1.** Summary of Peek-powered programmes starting in 2022.

Country	Programme(s)	Eligibility criteria *	Population	Time period
Botswana	National School Programme: ‘Pono Yame School Eye Health programme’	Every school child. Ages 5–18 years	500k	Feb 2022 – Feb 2025
Kenya	Ten counties with school and/or community eye health programmes	School children and/or community members	~10m	Jan 2022 – Jan 2025
Nepal	Community-based programme run by NNJS	Community members in the catchment area	~10k	July 2022 – July 2025
India	Regional programme in northern states of India. Study to be conducted within two screening programmes in Uttar Pradesh	Community members in the catchment area	~500k	2022 – July 2025

NNJS: Nepal Netra Jyoti Sangh *note: eligibility criteria are set locally for each screening programme

All participants currently provide their age, sex, language, and location, but no data on religion, ethnicity, income, education, or occupation. At the point of screening in the community, the screening software automatically captures data on their visual acuity, any diagnoses made, referral, and attendance status for treatment.

Although programmes are tailored to each context, there are a core set of stages: the participants’ first interaction with programme providers is when they are screened by a trained health or lay worker equipped with a hand-held android smartphone or tablet using the Peek Capture application. Participants have their vision assessed using the digitised and validated ‘tumbling Es’ approach ^11^. Those whose vision does not meet a pre-set threshold ‘screen positive’ and are referred to triage (which may be co-located with the screening operation, or may be performed at another time/place). For example, in some school-based programmes, triage happens in a room next door immediately after visual acuity screening. Whereas in some door-to-door community-based programmes, those who screen positive are referred on to attend the local primary care facility a week or so later. The most common service design involves referral to a triage clinic at a later time and in a different location.

At triage, all those who have screened positive are formally assessed by a more highly skilled cadre within the programme. Participants are either deemed to have normal vision (i.e. false positive screening), or they are diagnosed with an eye condition (‘triaged positive’). All of these true positive cases are treated with eye drops, other basic medications or provided with spectacles, as appropriate, at the community-based triage facility, or they are referred on for further specialist ophthalmic care (e.g. cataract surgery) if this cannot be provided on-site. This specialist care is often delivered at local hospitals. According to unpublished internal Peek data, up to half of those who screen positive and are referred on to triage actually attend their appointment. Even when triage is co-located with screening and happens on the same day, not everyone who screens positive attends. Furthermore, typically less than half of those referred on from triage for further ophthalmic assessment and treatment (e.g. spectacles or cataract surgery) attend. As part of a research grant to develop new approaches for continuous, equity-focused improvement, eye health programmes in Botswana, India, Kenya, and Nepal intend to start collecting, analysing, and reporting data on a wider range of sociodemographic variables, starting with new programmes launching in 2022. The same approach will be implemented for the further programmes that are planned in other countries. The intention is to identify the sociodemographic groups that are least likely to attend clinic, and then engage with representatives of these groups to explore potential service adaptations that could remove barriers and boost attendance rates, promoting proportionate universalism.

This work is being supported by academics, ministry of health officials, and health systems leaders in Botswana, India, Kenya, Nepal, and the UK.

### Objective

Our primary objective is to quantify the association between each sociodemographic domain and clinic attendance in order to establish which characteristics are most strongly associated with non-attendance.

### Research question

Of those diagnosed with an eye problem and referred on for treatment, which sociodemographic characteristics are most strongly associated with clinic non-attendance?

### Hypothesis

Non-attendance will be highest among marginalised groups; including unmarried or widowed women and girls, and those with the lowest levels of education, the fewest material assets, and no formal employment.

## Methods

### Study design

We will use an embedded, pragmatic cross-sectional design. Working with national Ministries of Health and implementing partner organisations, we will develop a list of sociodemographic questions that will be embedded within the screening process in each country and asked of every person identified with an eye problem and referred on for further ophthalmic assessment and/or care.

### Setting

The same cross-sectional design will be used to analyse routinely collected data from Peek-powered eye health programmes operating in Botswana, India, Kenya and Nepal. Peek runs a combination of community- and school-based programmes. Data collection will commence in 2022–2023. 

### Participants

This is an embedded, pragmatic study - as such data will be collected from the first 3,850 consecutive consenting adults and/or their children who present to Peek powered screening programmes in each study setting. Eligibility criteria are determined by national governments and local implementing partners. There are no exclusion criteria, however the youngest invitees are five years old. Parents/guardians will consent on behalf of their children.

### Data collection

When participants present to eye screening programmes they will be checked-in by programme implementers using the Peek Capture app running on android smartphones. Age, sex, phone ownership, and location data will be obtained for every participant at the point at which they enter the screening programmes.

The sociodemographic questions will be asked of all those who are identified with an eye problem at screening and referred to triage clinics. These data will form the basis of the analysis: exploring the proportion of those with each characteristic who do or do not attend their triage appointment in each country.

At both stages, programme implementers will enter responses using pre-set drop-down options. All those involved in data collection will receive standardised training from Peek Vision, including training on maintaining confidentiality and supporting respondents if they become distressed. The questions will be administered in a confidential setting.

These sociodemographic questions will be routinely used in all future Peek Vision-based screening programmes, however we will only publish data from consenting participants.

### Loss to follow up

Data of patients who are lost to follow-up will be kept and will be included in analysis as appropriate. Any study participant who wishes to withdraw from the trial will be initially counselled. If they still wish to withdraw from the study, consent will be requested to include the previously collected data in the analysis. If this is not given all data relating to that participant will be removed from the analysis and will be reported to the trial Data Safety and Monitoring Committee (DSMC).

### Closure

Recruitment into the study analysis will stop after 3,850 people have been enrolled, or 365 days after commencement in each country – whichever comes later. The one-year time period aligns with planned programme review meetings. As this study is a secondary analysis of (what will become) routinely collected data, there will be no changes to the way the screening programmes and their data collection approaches operate once the study ends.

Participants who entered the screening programme within three weeks of the end of the programme will be excluded from analysis of attendance rates. Those who were referred for treatment over three weeks before the close of the programme and had not attended by the close will be deemed non-attenders even if they subsequently attend after the closure of the study. This three-week cut-off is based on previous work from Kenya evincing a marked inflection point in attendance at this point.

### Variables

***Variable selection process***

**1. Literature review:** We started with a literature review to identify which variables are recommended by global health organisations. Many different sociodemographic indicators can and have been used to stratify population outcomes, and it seems that there is no international consensus around which are the most important to include. The WHO *World Report on Vision* and the recent *Lancet Global Health Commission on Global Eye Health* both call for international eye health data (including disease prevalence and care coverage) to be stratified by equity dimensions but do not specify which domains should be employed ^7, 12^.

Acknowledging the risk that “poorer, less advantaged segments of the population could be left behind” as countries expand access to health services, joint WHO and World Bank guidance recommends that managers gather data on gender, place of residence (urban/rural) and household income, expenditure, or wealth to allow comparisons between the rich and poor ^13^. The 2021 UN Resolution ‘Vision for Everyone’ highlights women and those living in poverty ^14^, and WHO’s *Making fair choices on the path to Universal Health Coverage* singles out “low-income groups and rural populations” ^15^. Finally, the WHO *Commission on Social Determinants of Health* identified: income, wealth, education, occupation, ethnicity/race/indigeneity, gender, area of living, refugee/immigrant status, sexual orientation, and religious and political beliefs ^6^. Galobardes and colleagues have previously developed a glossary of socioeconomic position indicators that provides the theoretical basis, measurement considerations, and strengths and limitations for many of these indicators ^16, 17^.

In total, our review identified the following 16 sociodemographic variables:

- Age
- Disability and other health conditions
- Education
- Ethnicity/race/colour/culture/indigenous group membership
- Gender
- Income/expenditure
- Location (urban/rural)
- Migrant/refugee/internally displaced status
- National origin
- Occupation
- Political beliefs
- Religion
- Sexual orientation
- Social capital
- Socioeconomic status
- Wealth/assets

Howe and colleagues have argued that sociodemographic metrics used in global development should be simple, reliable, reproducible, and linked to a well-understood social stratification process ^18^. Questions on age, gender, residence, ethnicity, marital status, education, and occupation all meet these criteria and have relatively non-contentious response options. However, it can be much more difficult to devise simple metrics to capture income, expenditure and wealth. We note the ubiquitous trade-off between comprehensiveness, fidelity, and feasibility here.

For this project we are primarily interested in the associations between attendance and individual sociodemographic characteristics rather than attendance and socioeconomic status. Socioeconomic status is a multidimensional construct that aims to capture access to resources and social position, often by combining income, education, and occupation ^16^.

**2. Secondary analysis of systematic review data:** Having established the variables recommended by the UN, WHO, World Bank, and Lancet Commission, next we assessed which domains are actually used in practice. We performed a secondary analysis of data from a concurrent systematic review that examines phone-based sociodemographic data collection in community-based health programmes. The full methods have been published elsewhere ^19^. This review included 11 studies that had tested different approaches to socioeconomic data collection using digital software. Data were collected from populations in eight countries (Australia, Bangladesh, Brazil, Burkina Faso, Kenya, the Netherlands, Tanzania and the USA). We assessed which variables were most commonly reported by these studies, taking an expansive view of sociodemographic variables that was grounded in work on the wider social determinants of health ^6, 20, 21^.

In total, 16 different variables were reported ( Table 2). At least a third of studies collected data on education, marital status, household income and employment status. None of the studies collected data on sexual orientation, religious or political beliefs, ethnicity or indigeneity. We postulate that this may be because of the stigma and social sensitivity surrounding these issues, however no information was provided on why these variables were omitted. We also note that whilst income and assets (housing type) were collected, none of the studies collected data on expenditure.

**Table 2.** Sociodemographic variables used in the included studies.

Domain	Number of studies
Education	9 (75%)
Marital status	5 (42%)
Employment status	4 (33%)
Household income	4 (33%)
Residence (urban/rural)	3 (25%)
Country of birth (immigrant status)	2 (17%)
Occupation	2 (17%)
Housing type	1 (8%)
Drug and alcohol use	1 (8%)
Household structure	1 (8%)
Local built infrastructure	1 (8%)
Parent's education	1 (8%)
Primary care registration	1 (8%)
Race	1 (8%)
School enrolment	1 (8%)
Wealth	1 (8%)

**3. Developing a master list of variables and indicators:** Based on our literature review and analysis of the systematic review data, we identified 11 broad domains that could feasibly be introduced into routine data collection processes in Peek powered programmes:

- Age
- Gender
- Residence (urban/rural)
- Language
- Ethnicity/tribe/race/caste
- Refugee/immigrant status
- Household structure: marital status for adults and parent/guardian status for children
- Religion
- Occupation
- Income
- Wealth

We drafted the initial response options to align with the USAID Demographic and Health Survey (DHS) model questionnaire ^22^ that has been used for over 400 surveys in 90 countries ^23^. and the Rapid Assessment of Avoidable Blindness (RAAB) instrument that has been used for more than 300 vision surveys in 79 countries ^24^. This was to ensure that all data will comply with international norms and can be maximally useful for domestic policymakers. We devised separate response options for adults and children (<18 years).

In large-scale screening programmes time is at a premium, as every additional question asked has a cumulative impact on the total number of people who can be screened each day. Furthermore, the Peek Vision screening app imposed a technical limitation in that only ten additional questions could be added to the existing screening flow. As previously noted, whilst there are rapid ways to ascertain age, gender, residence, language, refugee/immigrant status, relationships, religion, and occupation, it can be much more difficult to devise simple metrics to capture robust information on income and wealth. The DHS model survey includes over 100 questions on wealth and income, including long lists of assets, modes of transport, cooking fuels etc. The Equity Tool group ^25^ have used *principle component analysis* to identify smaller question sets that can be used to identify the poorest households in over 60 countries. However, these compressed question sets still involve asking more than ten questions, some of which have multiple choice answers. During an initial online workshop our team agreed that we would aim to ask 3–5 short and simple additional sociodemographic questions that would help us to distinguish between richer and poorer households in each country. The first draft of our master survey is presented in Table 3 below.

**Table 3.** Master survey - first draft – based on an online workshop discussion.

Domain (Data type)	Adult response options	Child response options	Notes
Age (years) (Discrete)	Any integer >18	Any integer 5 - 17	Already routinely collected in all Peek programmes
Gender (Categorical)	• Female • Male • Other	• Female • Male • Other	Already routinely collected in all Peek programmes The DHS and RAAB7 surveys only include female/ male. We have added ‘other’
Phone ownership (Ordinal)	Do you need someone else to receive your text message reminders? • Yes, my mother or father • Yes, my spouse • Yes, my daughter or son • Yes, other • No (= phone ownership)	Provided contact number: • My mother or father • My guardian • My teacher • Other	Already routinely collected in all Peek programmes
Place of residence (Categorical)	N/A	N/A	Urban/rural location automatically inferred from screening location
Distance from screening location to triage clinic (km) (Discrete)	N/A	N/A	Referred participants are given an appointment at a specific clinic Distance between screening location and triage clinic location has been found to be a predictor of outcomes This is automatically calculated by the Peek software.
Language (Categorical)	• [list languages]	• [list languages]	Country-specific lists will be derived from the latest Demographic and Health Survey
Relationships (Categorical)	• Married or living together • Divorced/separated • Widowed • Never married or lived together	Do you live with: • Both parents • Just one parent • Another relative or carer	Options may need tailoring depending on the context.
Ethnicity (Categorical)	• [List ethnic groups] • Other	• [List ethnic groups] • Other	Country-specific lists will be derived from the latest Demographic and Health Survey
Migrant/refugee (binary)	Are you a migrant or refugee? • Yes • No	Were your parents born in this country? • Yes • No	May be inflammatory depending on the setting
Religion (Categorical)	• [List main religions] • Other not listed • None	• [List main religions] • Other not listed • None	Country-specific lists will be derived from the latest Demographic and Health Survey
Education (Ordinal)	• None/pre-school only • Non-formal (included Quranic) • Some primary • Completed primary • Some secondary • Completed secondary • University	N/A – all participants will be in school	Options taken from the RAAB7 survey as it offers more detail than the DHS model questionnaire (early childhood education programme/Primary/ Secondary/Higher) Non-formal/Quranic options may not be appropriate in settings where the prevalence of these forms is negligible. All national child eye screening programmes in our study sites are implemented as school-based programmes
Occupation (Ordinal)	• Unemployed • Unskilled manual • Skilled manual • Professional • Homemaker	What are your parents’ jobs? • No parents • Unemployed • Unskilled work • Skilled work	For children, programme implementers will ask what their parent’s do for work and then code the highest occupational category on their behalf
Income (proxy) (Ordinal)	When you think about the food in your household would you say you have: • Less than adequate food for the needs of the household • Just adequate • More than adequate	When you think about the food in your household would you say you have: • Less than adequate food for the needs of the household • Just adequate • More than adequate	This question is being used in the RAAB7 eye health survey as a proxy for income The survey is designed for >50y olds, so the response options may not be appropriate for children
Income adequacy (Ordinal)	When you think about the income in your household would you say it is: • Not enough to cover our needs, we must borrow, • Not enough to cover our needs, we use savings, • Just enough to cover our needs, • Enough to cover our needs, we are able to save a little • Enough to cover our needs, we are building savings	When you think about the income in your household would you say it is: • Not enough to cover our needs, we must borrow • Just enough to cover our needs • Enough to cover our needs • More than enough, we are able to save	This question is being used in the RAAB7 eye health survey as a proxy for income The survey is designed for >50y olds, so the response options may not be appropriate for children
Wealth (Binary)	Is your house’s floor made out of cement? • Yes • No	Is your house’s floor made out of cement? • Yes • No	The specific indicator used here will depend on the location
Assets (Binary)	Does your household own: • [List assets from DHS]	Does your household own: • [List assets from DHS]	Shortest possible list of assets to be selected by country working groups

**Note**: Every question will have the additional options: ‘Do not want to answer’ and ‘Don’t know’.

**4: Tailoring surveys for individual countries:** Next we set up multistakeholder workshops in Botswana, India, Kenya and Nepal – the four countries where data collection will be embedded first – to review the internal and external validity of the domains for each sociocultural setting; tailor the response options; and identify the most appropriate assets to use in order to distinguish richer from poorer households. For each workshop we invited a LSHTM public health researcher, a representative from Peek Vision who lives/works in the country, a representative from at least one implementing partner (the organisations that conduct the data collection and screening in the field), and local academics with experience and expertise in sociodemographic data collection. The participants discussed each domain with reference to previous domestic data collection exercises, cultural attitudes, and the most recent national DHS ^26– 28^. The updated survey items were then reviewed with a health economists trained in socioeconomic assessment, sent for wider team input via email, and revised based on this feedback. The first draft socioeconomic surveys are presented in in Table 4– Table 7.

**Table 4.** Botswana sociodemographic questions following the multistakeholder workshop.

Domain	Adult response options	Child response options	Notes
Age	Any integer >18	Any integer 5 - 17	Already routinely gathered
Gender	• Female • Male • Other	• Female • Male • Other	Already routinely gathered
Phone ownership	Do you need someone else to receive your text message reminders? • Mother or father • Spouse • Daughter or son • Other • No (= phone ownership)	Provided contact number: • Mother or father • Guardian • Teacher • Other	Already routinely gathered
Place of residence	N/A	N/A	Urban/rural automatically inferred
Distance to clinic	N/A	N/A	Automatically calculated by Peek
Language	What language do you speak most often at home? • Setswana • English • Kalanga • Shekgalagari • Herero • Sebirwa • Mbukushu • Sesarwa • Shona • Ndebele • Setswapong • Afrikaans • Subiya • Shiyeyi • Other (specify)	What language do you speak most often at home? • Setswana • English • Kalanga • Shekgalagari • Herero • Sebirwa • Mbukushu • Sesarwa • Shona • Ndebele • Setswapong • Afrikaans • Subiya • Shiyeyi • Other (specify)	Categories taken from the Botswana 2017 DHS
Tribe	Which tribe do you originate from? • Tswana (or Setswana) • Kalanga • Basarwa • Kgalagadi • European • Other • Not sure	Do you know which tribe you originate from? • Tswana (or Setswana) • Kalanga • Basarwa • Kgalagadi • European • Other • Not sure	Workshop participants felt that it might be difficult to appropriately word a question about tribe/ethnicity and that this question. We note that the 2017 DHS does not ask about ethnicity or tribe.
Relationships	• Married • Never Married • Living Together • Separated • Divorced • Widowed	Do you live with: • Both parents • Just one parent • Another relative or carer	Workshop participants felt that we should separate married and living together into different options. Ideally, we would ask children if one or more parent had died, but we don’t want to cause distress. In the future we could consider asking teachers for this information
Migrant status	Are you a Botswana citizen? • Yes • No • Don’t want to answer	Were your parents born in this country? • Yes • No • Not sure	This is a sensitive question that adults may not want to answer [4% of the population is non-Batswana]
Religion	What is your religion? • Christian • Islam • Bahai • Hinduism • Badimo • Other	What is your religion? • Christian • Islam • Bahai • Hinduism • Badimo • Other	Options taken from the 2017 DHS
Education	What is you highest level of completed schooling? • Pre-school • Primary • Secondary • Tertiary • Non-formal education	N/A	All children will be in school Adult responses aligned with the Botswana 2017 DHS
Occupation	What is your occupation? • Unemployed • Unskilled manual • Skilled manual • Professional • Homemaker	What are your parents’ jobs? • [No parents] • Unemployed • Unskilled manual • Skilled manual • Professional • Homemaker	Interviewer to categorise and code the highest
Income (proxy)	When you think about the food in your household would you say you have: • Less than adequate food for the needs of the household • Just adequate • More than adequate	Did you eat yesterday before you sleep? • Yes • No Or How many times did you go to bed hungry last week (because there was no food)?	Question taken from RAAB7 – may remove due to poor face validity
Income adequacy	When you think about the income in your household would you say it is: • Not enough to cover our needs, we must borrow, • Not enough to cover our needs, we use savings, • Just enough to cover our needs, • Enough to cover our needs, we are able to save a little • Enough to cover our needs, we are building savings	Does your family receive food baskets or free school uniforms from social services? • Yes • No • Not sure	May want to re-phrase ‘social services’
Housing	Is your house’s floor made of cement or tiles? • Yes • No Where do you get water? • Piped indoors • Tap in the yard • Communal tap • Other What do you use for lighting? • Electricity • Paraffin • Candle • Solar • Wood What kind of toilet do you use at home? • Own flush toilet • Own latrine • Shared toilet/latrine • None	Is your house’s floor made of cement or tiles? • Yes • No Where do you get water? • Piped indoors • Tap in the yard • Communal tap • Other What do you use for lighting? • Electricity • Paraffin • Candle • Solar • Wood What kind of toilet do you use at home? • Own flush toilet • Own latrine • Shared toilet/latrine • None	8% of floors were made of mud and/or dung in 2017 Botswana is committed to ensuring availability and access to clean and safe water to its people (SDG6) All options taken from the 2017 DHS
Assets	Do you own a smartphone? • Yes • No Does your household own: • Bicycle • Motorcycle/scooter • Car or truck	Does your household own a smartphone like this? [hold up touchscreen phone] • Yes • No Does your household own: • Bicycle • Motorcycle/scooter • Car or truck	All options taken from the 2017 DHS

**Table 5.** Kenya sociodemographic questions following the multistakeholder workshop.

Domain	Adult response options	Child response options	Notes
Age	Any integer >18	Any integer 5 - 17	Already routinely gathered
Gender	• Female • Male • Other	• Female • Male • Other	Already routinely gathered
Phone ownership	Do you need someone else to receive your text message reminders? • Mother or father • Spouse • Daughter or son • Other • No (= phone ownership)	Provided contact number: • Mother or father • Guardian • Teacher • Other	Already routinely gathered
Place of residence	N/A	N/A	Urban/rural automatically inferred
Distance to clinic	N/A	N/A	Automatically calculated by Peek
Language	What language do you speak most often at home? • English • Swahili • Borana • Embu • Kalenjin • Kamba • Kikuyu • Kisii • Luhya • Maragoli • Luo • Maasai • Meru • Mijikenda • Pokot • Somali • Turkana • Other	What language do you speak most often at home? • English • Swahili • Borana • Embu • Kalenjin • Kamba • Kikuyu • Kisii • Luhya • Maragoli • Luo • Maasai • Meru • Mijikenda • Pokot • Somali • Turkana • Other	Workshop participants felt that it would be inflammatory to ask about tribe/ethnicity. Language will be used as a proxy
Relationships	• Never married • Married • Living together • Single • Divorced/separated • Widowed	*Do you live with:* • Both parents • Just one parent • Another relative • Guardian (non-relative) • Orphanage	Ideally, we would ask children if one or more parent had died, but we don’t want to cause distress. In the future we could consider asking teachers for this information
Migrant status	Were you born in Kenya? • Yes • No • Don’t want to answer	Were your parents born in this country? • Yes • No • Not sure	This question may be redundant. Kenya is currently home to 500,000 refugees, however they mainly live in camps and this information will already be collected under ‘place of residence’. Outside of Nairobi, the migrant population that does not live in camps is negligible.
Religion	What is your religion? • Roman Catholic • Protestant/other Christian • Islam • Other • No religion	What is your religion? • Roman Catholic • Protestant/other Christian • Islam • Other • No religion	Responses taken from the 2014 DHS
Education	What is you highest level of completed schooling? • No education • Some primary • Primary complete • Some secondary • Secondary complete • More than secondary	N/A	All children will be in school Adult responses aligned with the 2014 DHS
Occupation	What is your occupation? • Unemployed • Agriculture • Unskilled manual • Skilled manual • Sales and services • Clerical • Professional/technical/managerial • Homemaker	What are your parents’ jobs? • No parents • Unemployed • Agriculture • Unskilled manual • Skilled manual • Sales and services • Clerical • Professional/technical/ managerial • Homemaker	Interviewer to categorise and code the highest
Income	When you think about the food in your household would you say you have: • Less than adequate food for the needs of the household • Just adequate • More than adequate	Did you go to bed hungry last night? • Yes • No Or How many times did you go to bed hungry last week (because there was no food)?	Question taken from RAAB7 – may remove due to poor face validity
Income adequacy	When you think about the income in your household would you say it is: • Not enough to cover our needs, we must borrow, • Not enough to cover our needs, we use savings, • Just enough to cover our needs, • Enough to cover our needs, we are able to save a little • Enough to cover our needs, we are building savings	N/A	From RAAB7, but poor face validity.
Housing	Is your house’s floor made of earth, sand, or dung? • Yes • No Do you have water piped into your own house or yard? • Yes • No Does your household have electricity? • Yes • No What kind of toilet does your household you use? • Own toilet/latrine • Shared toilet/latrine • None (bush/field)	Is your house’s floor made of earth, sand, or dung? • Yes • No Do you have water piped into your own house or yard? • Yes • No Does your household have electricity? • Yes • No What kind of toilet does your household you use? • Own toilet/latrine • Shared toilet/latrine • None (bush/field)	All options taken from the 2014 DHS
Assets	Do you own a smartphone? • Yes • No Does your household own a: • Bicycle • Motorcycle/scooter • Car or truck Do you own your dwelling? • Yes • No	Does your household own a smartphone? • Yes • No Does your household own a: • Bicycle • Motorcycle/scooter • Car or truck	

**Table 6.** Nepal sociodemographic questions following the multistakeholder workshop.

Domain	Adult response options	Child response options	Notes
Age	Any integer >18	Any integer 5 - 17	Already routinely gathered
Gender	• Female • Male • Other	• Female • Male • Other	Already routinely gathered
Phone ownership	Do you need someone else to receive your text message reminders? • Mother or father • Spouse • Daughter or son • Other • No (= phone ownership)	Provided contact number: • Mother or father • Guardian • Teacher • Other	Already routinely gathered
Place of residence	N/A	N/A	Urban/rural automatically inferred
Distance to clinic	N/A	N/A	Automatically calculated by Peek
Language	What language do you speak most often at home? • Nepali • Maithali • Bhojpuri • Tharu • Tamang • Newar • Bajjika • Magar • Doteli • Urdu • Avadhi • Limbu • Gurung • Baitadeli • Other	What language do you speak most often at home? • Nepali • Maithali • Bhojpuri • Tharu • Tamang • Newar • Bajjika • Magar • Doteli • Urdu • Avadhi • Limbu • Gurung • Baitadeli • Other	
Ethnicity	What is your ethnicity? • Hill Brahmin • Hill Chhetri • Terai Brahmin/Chhetri • Other Terai caste • Hill Dalit • Terai Dalit • Newar • Hill Janajati • Terai Janajati • Muslim • Migrant • Other	What is your ethnicity? • Hill Brahmin • Hill Chhetri • Terai Brahmin/Chhetri • Other Terai caste • Hill Dalit • Terai Dalit • Newar • Hill Janajati • Terai Janajati • Muslim • Migrant • Other	Responses taken from 2016 DHS
Relationships	*What is your current marital status?* • Never married • Married • Divorced/separated • Widowed Has your partner been living away for the past six months or more? • Yes • No	*Do you live with:* • Both parents • Just one parent • Another relative • Guardian (non-relative) • Orphanage	One third of married couples live apart Options taken from the 2016 DHS Ideally, we would ask children if one or more parent had died, but we don’t want to cause distress. In the future we could consider asking teachers for this information The Nepal team wanted a specific question asking if children not living with their parents are orphans
Migrant status	Were you born in Nepal? • Yes • No • Don’t want to answer	Were your parents born in this country? • Yes • No • Not sure	
Religion	What is your religion? • Hindu • Buddhist • Muslim • Kirat • Christian • No religion • Other	What is your religion? • Hindu • Buddhist • Muslim • Kirat • Christian • No religion • Other	Responses taken from the 2016 DHS
Education	What is you highest level of completed schooling? • No education • Primary • Some secondary • SLC and above (‘school leaving certificate’)	N/A	All children will be in school Adult responses taken from the 2016 DHS
Occupation	What is your occupation? • Unemployed • Agriculture • Unskilled work • Government or private employee • Business owner / professional • Housewife	What is your father’s job? • Unemployed / no father • Agriculture • Unskilled work • Government or private employee • Business owner / professional	Interviewer to categorise and code the highest
Income	When you think about the food in your household would you say you have: • Less than adequate food for the needs of the household • Just adequate • More than adequate	Did you go to bed hungry last night? • Yes • No Or How many times did you go to bed hungry last week (because there was no food)?	Question taken from RAAB7 The team feel this question has poor face validity – to be discussed further with another health economist
Income adequacy	When you think about the income in your household would you say it is: • Not enough to cover our needs, we must borrow, • Not enough to cover our needs, we use savings, • Just enough to cover our needs, • Enough to cover our needs, we are able to save a little • Enough to cover our needs, we are building savings	N/A	To be discussed with another health economist – the team are not convinced this is a good measure
Housing	Is your house’s floor made of cement? • Yes • No Do you have water piped into your own house or yard? • Yes • No Does your household have electricity? • Yes • No What kind of toilet does your household you use? • Own toilet/latrine – inside dwelling • Own toilet/latrine – in yard/plot • Shared toilet/latrine • None (bush/field)	Is your house’s floor made of cement? • Yes • No Do you have water piped into your own house or yard? • Yes • No Does your household have electricity? • Yes • No What kind of toilet does your household you use? • Own toilet/latrine – inside dwelling • Own toilet/latrine – in yard/plot • Shared toilet/latrine • None (bush/field)	All options taken from the 2016 DHS
Assets	Do you own a smartphone? • Yes • No Does your household own a: • Bicycle or rickshaw • Motorcycle or scooter • Car or truck • Three-wheel tempo Do you own your dwelling? • Yes • No	Does your household own a smartphone? • Yes • No Does your household own a: • Bicycle or rickshaw • Motorcycle or scooter • Car or truck • Three-wheel tempo	

**Table 7.** India sociodemographic questions following the multistakeholder workshop.

Domain (Data type)	Adult response options	Child response options	Notes
Age (years) (Discrete)	Any integer >18	Any integer 5 - 17	Already routinely collected in all Peek programmes
Gender (Categorical)	• Female • Male • Other	• Female • Male • Other	Already routinely collected in all Peek programmes The DHS and RAAB7 surveys only include female/male. We have added ‘other’
Phone ownership (Ordinal)	Do you need someone else to receive your text message reminders? • Yes, my mother or father • Yes, my spouse • Yes, my daughter or son • Yes, other • No (= phone ownership)	Provided contact number: • Yes, my mother or father • Yes, my guardian • Yes, my teacher • Yes, other	Already routinely collected in all Peek programmes
Place of residence (Categorical)	N/A	N/A	Urban/rural location automatically inferred from screening location
Distance from screening location to triage clinic (km) (Discrete)	N/A	N/A	Referred participants are given an appointment at a specific clinic Distance between screening location and triage clinic location has been found to be a predictor of outcomes This is automatically calculated by the Peek software.
Language (Categorical)	• [list languages]	• [list languages]	Mostly Hindi, but there are a few other dialects
Relationships (Categorical)	• Married or living together • Divorced/separated • Widowed • Never married or lived together	Do you live with: • Both parents • Just one parent • Another relative or carer	Options may need tailoring depending on the context.
Ethnicity (Categorical)	• [List ethnic groups] • Other	• [List ethnic groups] • Other	Country-specific lists will be derived from the latest Demographic and Health Survey
Migrant/refugee (binary)	Are you a migrant or refugee? • Yes • No	Were your parents born in this country? • Yes • No	May be inflammatory depending on the setting
Religion (Categorical)	• [List main religions] • Other not listed • None	• [List main religions] • Other not listed • None	Country-specific lists will be derived from the latest Demographic and Health Survey
Education (Ordinal)	• Professional degree • Graduate or postgraduate • Intermediate or post high school diploma • High school certificate • Middle school certificate • Primary school certificate • Illiterate	N/A – all participants will be in school	Options aligned with local authority options. All national child eye screening programmes in our study sites are implemented as school-based programmes
Occupation (Ordinal)	• Professional (white collar) • Semi-professional • Clerical/ shop-owner/ farm • Skilled worker • Semi-skilled worker • Unskilled worker • Unemployed	What are your parents’ jobs? • No parents • Unemployed • Unskilled work • Skilled work	For children, programme implementers will ask what their parent’s do for work and then code the highest occupational category on their behalf
Income (proxy) (Ordinal)	• less than 6k • 7k-10k • greater than 10k,	• N/A	Thresholds discussed with health economist
Wealth (Binary)	Is your house’s floor made out of cement? • Yes • No	Is your house’s floor made out of cement? • Yes • No	The specific indicator used here will depend on the location
Assets (Binary)	Does your household own: • [List assets from DHS]	Does your household own: • [List assets from DHS]	Shortest possible list of assets to be selected by country working groups

**5. Whole-team in-person workshop**

In February 2022, the research collaborators from Kenya, Botswana and Nepal met in Nairobi to review the survey questions with academics, three health economists, and in-country implementing partners. A series of interactive sessions were held to review the underlying literature, revisit the intended outcomes, and examine recent survey approaches used in each country. Individual country teams then honed the domain list and question response items with support from the wider research collaborators. The final sociodemographic surveys for each country are presented below in Table 8– Table 10. The India screening programme team did not come to Nairobi. They will refine their survey items using a series of online workshops.

**Table 8.** Botswana update from in-person February workshop. To be translated into Setswana.

Domain	Adult response options	Child response options	Notes
Age	Any integer >18	Any integer 5 - 17	Already routinely gathered
Gender	• Female • Male • Other	• Female • Male	Already routinely gathered ‘Other’ removed for children as it is controversial and does not align with national data collection practices
Phone ownership	Do you need someone else to receive your text message reminders? • Mother or father • Spouse • Daughter or son • Other • No (= phone ownership)	Provided contact number: · Mother or father · Guardian · Teacher · Other	Already routinely gathered
Place of residence	N/A	N/A	Urban/rural automatically inferred
Distance to clinic	N/A	N/A	Automatically calculated by Peek
Language	What language do you speak most often at home? • Setswana • English • Kalanga • Shekgalagari • Herero • Mbukushu • Sesarwa • Shona • Ndebele • Afrikaans • Subiya • Shiyeyi • Other (specify)	What language do you speak most often at home? • Setswana • English • Kalanga • Shekgalagari • Herero • Mbukushu • Sesarwa • Shona • Ndebele • Afrikaans • Subiya • Shiyeyi • Other (specify)	Setswapong and Sebirwa were removed from the original list as they are not different from Setswana
Tribe	Which tribe do you originate from? • Setswana • Kalanga • Shekgalagari • Herero • Mbukushu • Sesarwa • Shona • Ndebele • Afrikaans • Subiya • Shiyeyi • Other (specify) • Not sure	Do you know which tribe you originate from? • Setswana • Kalanga • Shekgalagari • Herero • Mbukushu • Sesarwa • Shona • Ndebele • Afrikaans • Subiya • Shiyeyi • Other (specify) • Not sure	Tribes have been aligned with languages. ‘English’ has been removed.
Relationships	• Married • Never Married • Living Together • Separated • Divorced • Widowed	Do you live with: • Your father • Mother • Grandparent(s) • Aunt • Uncle • Siblings • Other	The options have been expanded to add a greater degree of specificity
Household	How many people live in your home? [number]	How many people live in your home? [number]	This question has been added because it may be an important predictor of attendance i.e. large families with low incomes may struggle to pay for transport costs
Migrant status	Are you a Botswana citizen? • Yes • No • Don’t want to answer	Were your parents born in Botswana? • Yes • No • Not sure	Changed ‘this country’ to ‘Botswana’ to be more specific
Religion	What is your religion? • Christian • Islam • Bahai • Hinduism • Badimo • Other	What is your religion? • Christian • Islam • Bahai • Hinduism • Badimo • Other	No changes made Options taken from the 2017 DHS
Education	What is you highest level of completed schooling? • Pre-school • Primary • Secondary • Tertiary • Non-formal education	N/A	No changes made Options align with the 2017 DHS
Disability	Do you have difficulty **hearing**, even if using a hearing aid(s)? • No difficulty • Some difficulty • A lot of difficulty • Cannot do at all • Don’t know Do you have difficulty walking or climbing steps? • No difficulty • Some difficulty • A lot of difficulty • Cannot do at all • Don’t know Do you have difficulty remembering or concentrating? • No difficulty • Some difficulty • A lot of difficulty • Cannot do at all • Don’t know Do you have difficulty with **self-care**, such as washing all over or dressing? • No difficulty • Some difficulty • A lot of difficulty • Cannot do at all • Don’t know Using your usual language, do you have difficulty **communicating**, for example understanding or being understood? • No difficulty • Some difficulty • A lot of difficulty • Cannot do at all • Don’t know	Do you have difficulty **hearing**, even if using a hearing aid(s)? • No difficulty • Some difficulty • A lot of difficulty • Cannot do at all • Don’t know Do you have difficulty walking or climbing steps? • No difficulty • Some difficulty • A lot of difficulty • Cannot do at all • Don’t know Do you have difficulty remembering or concentrating? • No difficulty • Some difficulty • A lot of difficulty • Cannot do at all • Don’t know Do you have difficulty with **self-care**, such as washing all over or dressing? • No difficulty • Some difficulty • A lot of difficulty • Cannot do at all • Don’t know Using your usual language, do you have difficulty **communicating**, for example understanding or being understood? • No difficulty • Some difficulty • A lot of difficulty • Cannot do at all • Don’t know	New question added at the request of implementing partners Response options taken from the Washington Group Short Set on Functioning: https://www.washingtongroup- disability.com/question-sets/wg- short-set-on-functioning-wg-ss/ The same options will be used for adults and children. UNICEF does have a child-specific question set, but it is more than double the length.
Occupation	What is your occupation? • Unemployed • Unskilled manual • Skilled manual • Professional • Homemaker	What are your parents’/guardian’s jobs? • [No parents] • Unemployed • Unskilled manual • Skilled manual • Professional • Homemaker	We have added ‘guardians’ Interviewer to categorise and code the highest
Income adequacy	When you think about the income in your household would you say it is: • Not enough to cover our needs, we must borrow, • Not enough to cover our needs, we use savings, • Just enough to cover our needs, • Enough to cover our needs, we are able to save a little • Enough to cover our needs, we are building savings	Does your family receive food baskets or free school uniforms from social workers? • Yes • No • Not sure	We removed the question on food sufficiency. We felt it was unlikely to render robust data Botswana is the only country that will retain the RAAB7 subjective question on income sufficiency We re-phrased ‘social services’ to ‘social workers’
Housing	Is your house’s floor made of cement or tiles? • Yes • No Where do you get water? • Piped indoors • Tap in the yard • Communal tap • Other What do you use for lighting? • Electricity • Paraffin • Candle • Solar • Wood What kind of toilet do you use at home? • Own flush toilet • Own latrine • Shared toilet/latrine • None	Is your house’s floor made of cement or tiles? • Yes • No Where do you get water? • Piped indoors • Tap in the yard • Communal tap • Other What do you use for lighting? • Electricity • Paraffin lamp (lebone) • Candle • Solar • Wood What kind of toilet do you use at home? • Flush toilet • Pit latrine • Shared toilet/pit latrine • None	All options taken from the 2017 DHS 8% of floors were made of mud and/ or dung in 2017 Botswana is committed to ensuring availability and access to clean and safe water to its people (SDG6) Added ‘lamp (lebone)’ for paraffin We removed ‘own’ for toilet as we felt this word is redundant We added ‘pit’ before latrine
Assets	Do you own a smartphone? • Yes • No Does your household own: • Bicycle • Motorcycle/scooter • Car or truck	Does your household own a smartphone like this? [hold up touchscreen phone] • Yes • No Does your household own: • Bicycle • Motorcycle/scooter • Car or truck	No changes made. All options taken from the 2017 DHS

**Table 9.** Kenya update from in-person February workshop. To be translated into Kiswahili.

Domain	Adult response options (>18y)	Child response options	Notes
Age	How old are you?	How old are you	Already routinely gathered
Gender	• Female • Male • Other	• Female • Male • Other	Already routinely gathered
Phone ownership	Do you need someone else to receive your text message reminders? • Mother or father • Spouse • Daughter or son • Other • No (= phone ownership)	Provided contact number: • Mother or father • Guardian • Teacher • Other	Already routinely gathered
Place of residence	N/A	N/A	Urban/rural automatically inferred
Distance to clinic	N/A	N/A	Automatically calculated by Peek
Language	What is your mother tongue? • English • Swahili • Borana • Embu • Kalenjin • Kamba • Kikuyu • Kisii • Luhya • Maragoli • Luo • Maasai • Meru • Mijikenda • Pokot • Somali • Turkana • Other	What is your mother tongue? • English • Swahili • Borana • Embu • Kalenjin • Kamba • Kikuyu • Kisii • Luhya • Maragoli • Luo • Maasai • Meru • Mijikenda • Pokot • Somali • Turkana • Other	‘What language do you speak most often at home?’ changed to ‘What is your mother tongue?’ as we felt this was more specific This will be used as a proxy for ethnicity
Relationships	• Married • Single • Divorced/separated • Widowed • Other	*Do you live with:* • Both parents • Just one parent • Another relative • Guardian (non-relative) • Orphanage	We removed ‘never married’ because this is the same as single We removed ‘living together’ because this question is loaded with social stigma Ideally, we would ask children if one or more parent had died, but we don’t want to cause distress. In the future we could consider asking teachers for this information
Religion	What is your religion? • Christian • Islam • Hindu • Other	What is your religion? • Christian • Islam • Hindu • Other	We removed ‘no religion’ as this group is negligible Christian denominations were aggregated, and we added ‘Hindu’
Education	What is you highest completed level of schooling? • No education • Primary • Secondary • Post-secondary	N/A	We reworded the question and removed ‘completed’ and ‘some’ options to simplify the list
Disability	Do you have difficulty **hearing**, even if using a hearing aid(s)? • No difficulty • Some difficulty • A lot of difficulty • Cannot do at all • Don’t know Do you have difficulty walking or climbing steps? • No difficulty • Some difficulty • A lot of difficulty • Cannot do at all • Don’t know Do you have difficulty remembering or concentrating? • No difficulty • Some difficulty • A lot of difficulty • Cannot do at all • Don’t know Do you have difficulty with **self-care**, such as washing all over or dressing? • No difficulty • Some difficulty • A lot of difficulty • Cannot do at all • Don’t know Using your usual language, do you have difficulty **communicating**, for example understanding or being understood? • No difficulty • Some difficulty • A lot of difficulty • Cannot do at all Don’t know	Do you have difficulty **hearing**, even if using a hearing aid(s)? • No difficulty • Some difficulty • A lot of difficulty • Cannot do at all • Don’t know Do you have difficulty walking or climbing steps? • No difficulty • Some difficulty • A lot of difficulty • Cannot do at all • Don’t know Do you have difficulty remembering or concentrating? • No difficulty • Some difficulty • A lot of difficulty • Cannot do at all • Don’t know Do you have difficulty with **self-care**, such as washing all over or dressing? • No difficulty • Some difficulty • A lot of difficulty • Cannot do at all •Don’t know Using your usual language, do you have difficulty **communicating**, for example understanding or being understood? • No difficulty • Some difficulty • A lot of difficulty • Cannot do at all Don’t know	New question added at the request of implementing partners Response options taken from the Washington Group Short Set on Functioning: https://www.washingtongroup- disability.com/question-sets/wg- short-set-on-functioning-wg-ss/ The same options will be used for adults and children. UNICEF does have a child-specific question set, but it is more than double the length.
Occupation	What is your occupation? • Not employed • Agriculture • Domestic service • Unskilled manual • Skilled manual • Sales and services • Clerical • Professional/technical/managerial	What are your parents’ jobs? [staff to categorise & code only the highest] • No parents • Not employed • Agriculture • Donestic services • Unskilled manual • Skilled manual • Sales and services • Clerical • Professional/technical/managerial	We aligned the occupation categories with the 2014 DHS, adding domestic services
Income	What income band are you in? • Less than 24,000 KSh/month (288,000/yr, 10% Tax band) • Bewteen 24,000 - 32,333 KSh/ month (288,000 - 100,000/yr, 25% Tax band) • More than 32,333 KSh/month (388,000/yr, 30% Tax band)	N/A	We removed the question on food adequacy as we felt it was not likely to render robust information We also dropped the subjective question on income adequacy due to concerns about face validity. We replaced these income questions with a more direct item on income categories, based on the Kenya Revenue Authority tax bands.
Housing	What is your floor made of in your house? • Cement • Other Do you have a source of water within your compound? • Yes • No Does your household have electricity, solar, or a generator? • Yes • No What type of toilet facility do members of your households usually use? • Own toilet/latrine • Communal toilet/latrine • None (bush/field)	What is your floor made of in your house? • Cement • Other Do you have a source of water within your compound? - Yes - No Does your household have electricity, solar, or a generator? • Yes • No What type of toilet facility do members of your households usually use? • Own toilet/latrine • Communal toilet/latrine • None (bush/field)	We switched from ‘earth, sand or dung’ to ‘cement’. This is the reciprocal question and is faster to ask. We switched from ‘do you have water piped into your own house or yard?’ to ‘do you have a source of water within your compound’ because some rich people use boreholes We revised the wording of the toilet question changed to add greater clarity All options are aligned with the 2014 DHS
Assets	Do you own a smartphone? • Yes • No Does your household own a: • Bicycle • Motorcycle/scooter • Car or truck • None • Other	Does your household own a smart phone (with a touch screen)? • Yes • No Does your household own a: • Bicycle • Motorcycle/scooter • Car or truck • None • Other	We noted that smartphone ownership is so prevalent that it is only a sensible proxy for wealth in rural areas

**Note** – The question on **migrant status** was removed on the basis that the migration population is negligible, and screening conducted in refugee camps will be signalled by location.

**Table 10.** Nepal – update from in-person February workshop. To be translated into Nepali and Maithali.

Domain	Adult response options	Child response options	Notes
Age	Any integer >18	Any integer 5 - 17	Already routinely gathered
Gender	• Female • Male • Other	• Female • Male • Other	Already routinely gathered
Phone ownership	Do you need someone else to receive your text message reminders? • Mother or father • Spouse • Daughter or son • Other • No (= phone ownership)	Provided contact number: • Mother or father • Guardian • Teacher • Other	Already routinely gathered
Place of residence	N/A	N/A	Urban/rural automatically inferred
Distance to clinic	N/A	N/A	Automatically calculated by Peek
Language	What language do you speak most often at home? • Nepali • Maithali • Bhojpuri • Tharu • Tamang • Newar • Bajjika • Magar • Doteli • Urdu • Avadhi • Limbu • Gurung • Baitadeli • Other	What language do you speak most often at home? • Nepali • Maithali • Bhojpuri • Tharu • Tamang • Newar • Bajjika • Magar • Doteli • Urdu • Avadhi • Limbu • Gurung • Baitadeli • Other	No changes
Ethnicity	What is your ethnicity? • Hill Brahmin • Hill Chhetri • Terai Brahmin/Chhetri • Other Terai caste • Hill Dalit • Terai Dalit • Newar • Hill Janajati • Terai Janajati • Muslim • Migrant • Other	What is your ethnicity? • Hill Brahmin • Hill Chhetri • Terai Brahmin/Chhetri • Other Terai caste • Hill Dalit • Terai Dalit • Newar • Hill Janajati • Terai Janajati • Muslim • Migrant • Other	No changes
Relationships	*What is your current marital status?* • Never married • Married • Divorced/separated • Widowed Has your partner been living away for the past six months or more? • Yes • No	*Do you live with:* • Both parents • Just one parent • Another relative • Guardian (non-relative) • Orphanage	No changes
Health insurance coverage	Do you have active medical health insurance today? - Yes - No	N/A	New question added for medical health tourists on the basis that many Indians cross the border to access care
Religion	What is your religion? • Hindu • Buddhist • Muslim • Kirat • Christian • No religion • Other	What is your religion? • Hindu • Buddhist • Muslim • Kirat • Christian • No religion • Other	No changes
Education	What is you highest level of completed schooling? • No formal education • Primary • Lower secondary • SLC or higher secondary • University	N/A	Adult options refined: ‘Some secondary’ changed to ‘Lower secondary’ and ‘SLC and above’ changed to ‘SLC or higher secondary’ or ‘university’
Disability	Do you have difficulty **hearing**, even if using a hearing aid(s)? • No difficulty • Some difficulty • A lot of difficulty • Cannot do at all • Don’t know Do you have difficulty walking or climbing steps? • No difficulty • Some difficulty • A lot of difficulty • Cannot do at all • Don’t know Do you have difficulty remembering or concentrating? • No difficulty • Some difficulty • A lot of difficulty • Cannot do at all • Don’t know Do you have difficulty with **self-care**, such as washing all over or dressing? • No difficulty • Some difficulty • A lot of difficulty • Cannot do at all • Don’t know Using your usual language, do you have difficulty **communicating**, for example understanding or being understood? • No difficulty • Some difficulty • A lot of difficulty • Cannot do at all Don’t know	Do you have difficulty **hearing**, even if using a hearing aid(s)? • No difficulty • Some difficulty • A lot of difficulty • Cannot do at all • Don’t know Do you have difficulty walking or climbing steps? • No difficulty • Some difficulty • A lot of difficulty • Cannot do at all • Don’t know Do you have difficulty remembering or concentrating? • No difficulty • Some difficulty • A lot of difficulty • Cannot do at all • Don’t know Do you have difficulty with **self-care**, such as washing all over or dressing? • No difficulty • Some difficulty • A lot of difficulty • Cannot do at all • Don’t know Using your usual language, do you have difficulty **communicating**, for example understanding or being understood? • No difficulty • Some difficulty • A lot of difficulty • Cannot do at all Don’t know	New question added at the request of implementing partners Response options taken from the Washington Group Short Set on Functioning: https://www.washingtongroup- disability.com/question-sets/wg- short-set-on-functioning-wg-ss/ The same options will be used for adults and children. UNICEF does have a child-specific question set, but it is more than double the length.
Occupation	What is your occupation? • Unemployed • Agriculture • Unskilled work • Government or private employee • Business owner / professional • Housewife	What is your father’s job? • Unemployed / no father • Agriculture • Unskilled work • Government or private employee • Business owner / professional	Father used on the basis that this is the best indicator of socioeconomic status. Women have more senior positions than their male partners in a negligible proportion of households Interviewer to categorise and code the highest
Income adequacy	When you think about the income in your household would you say it is: • Not enough to cover our needs, we must borrow, • Not enough to cover our needs, we use savings, • Just enough to cover our needs, • Enough to cover our needs, we are able to save a little • Enough to cover our needs, we are building savings	N/A	No changes
Housing	Is your house’s floor made of cement? • Yes • No Do you have water piped into your own house or yard? • Yes • No Does your household have electricity? • Yes • No What kind of toilet does your household you use? • Own toilet/latrine – inside dwelling • Own toilet/latrine – in yard/plot • Shared toilet/latrine • None (bush/field)	Is your house’s floor made of cement? • Yes • No Do you have water piped into your own house or yard? • Yes • No Does your household have electricity? • Yes • No What kind of toilet does your household you use? • Own toilet/latrine – inside dwelling • Own toilet/latrine – in yard/plot • Shared toilet/latrine • None (bush/field)	No changes
Assets	Do you own a smartphone? • Yes • No Does your household own a: • Bicycle or rickshaw • Motorcycle or scooter • Car or truck • Three-wheel tempo Do you own your dwelling? • Yes • No	Does your household own a smartphone? • Yes • No Does your household own a: • Bicycle or rickshaw • Motorcycle or scooter • Car or truck • Three-wheel tempo	No changes

Notes: The question on migrant status (‘Were you born in Nepal’) was removed as the team felt it will be too inflammatory. The Nepalese team were concerned that data collectors will not have capacity to ask all of the questions. If this turns out to be the case, the team feel that Income, Occupation, and Housing are the three most important indicators to focus on.

***Summary of major changes from the in-person workshop:*** Migrant status was dropped from the Kenyan and Nepalese surveys. Household composition was added in Botswana. Disability was included in all settings. Nepal added a question on whether adults had health insurance. The income questions changed in all settings, and the food adequacy question was dropped for all settings. Workshops to refine the question list are still to take place in India.

***6. Translation and piloting:*** The finalised survey instruments will be translated into the most commonly spoken language in each setting, back-translated into English to check that meaning has not been lost, and then piloted with laypeople by a domestic research assistant using a ‘think aloud’ approach ^29^. Further refinements to optimise the response options and wording based on this feedback will be incorporated and re-translated

***7. Post-pilot review:*** After the first six months we will review the questions with the data collectors and ask:

- Do any of the questions require clarifications? How could they be re-worded?
- Were any questions problematic, inappropriate, or particularly sensitive? How could they be reworded?
- Where there any questions where the interviewer felt the participants were not answering accurately?
- Do any questions need to be dropped? Or added?
- Do the questions perform adequately for all age groups?
- Is the time taken to ask the additional questions appropriate?

We will analyse the sociodemographic data that have been collected by programme implementers using the Peek Acuity app to calculate the mean number of seconds spent on each question, along with the interquartile range. We will discuss potential ways to reduce the time spent on the longest questions with the broader team. We will also assess the data entry information to assess which questions are most likely to be skipped, and whether the responses for any particular questions display characteristics that signal gaming or misinterpretation e.g., the first response option being ticked disproportionately often. Given that our questions draw heavily on the DHS model survey that is designed for >15 year olds, we will also perform stratified analyses that examine response rates and rates of missing items by age band. We will assess the validity and reliability of the questionnaire.

### Outcome measures

Our primary outcome is attendance at triage clinic within 21 days (including weekends) of referral. We will compare this outcome between categories of sociodemographic characteristic. This cut-off has been selected because most appointments are made for one week after triage, and very few people attend more than two-weeks after their appointed date. Attendance is routinely recorded in the Peek Capture app when participants check-in to the clinic. Participants who entered the screening programme within three weeks of the end of the programme will be excluded from analysis of attendance rates. Those who were referred over three weeks before the close of the programme and had not attended by the close will be deemed non-attenders (even if they subsequently attend after the closure of the study).

### Secondary analyses

We within 21 days of being referred from the triage clinic will report the prevalence of vision impairment among those presenting to triage, by sociodemographic group.

A secondary outcome is attendance at the ophthalmic clinic within 21 days.

***Sample size and data collection***

Set against the ubiquitous constraint of limited resources, the central principle that drives sample size calculations is clinical significance i.e. when does a difference in attendance between two groups become important? We may feel that 2% higher attendance among men compared to women is not particularly concerning but a 20% difference represents a major equity issue. The decision about where the threshold of importance lies ultimately comes down to values, and the process of settling on a meaningful maximum margin of error is a value judgement. We held online deliberative discussions in each country with input from lay representatives. In all sites we agreed that 5–10% differences between groups represent what we felt to be the lower bound of ‘significant’. As such, we will use a 5% margin of error.

We are aiming to compare odds of attendance between sociodemographic subgroups. The number of people in each subgroup is the factor that will determine our ability to make statistically significant comparisons for a given level of α, as well as determining the probability of type II error (i.e. incorrectly rejecting the null hypothesis). With a 95% confidence level, a 5% margin of error, and a maximally conservative proportion of 0.5, we would need to have at least 385 people in each subgroup to make statistically significant comparisons between groups.

In each study site we will collect data from the first 3,850 consecutive consenting people who are referred. This will enable us to make statistically significant comparisons between groups that contain at least 10% of the overall population. Data will be collected by screening staff/volunteers working in each setting as part of the routine screening process that is conducted using the Peek Vision app.

***Statistical analysis***

**Statistical methods**

We will use logistic regression to report odds ratios for the outcome (non-attendance) for each sociodemographic domain in each country. Complete case analyses will be performed initially, but if missing data are more than 5% for a given variable then we will perform a range of sensitivity analyses to check the robustness of our estimates. We will publish anonymised aggregate data online, along with all our statistical code.

Our primary aim is identifying the population subgroups with the lowest attendance so that we can ultimately engage with representatives of these groups to try and improve access in future work. We will use multivariable logistic regression to calculate the adjusted odds of attendance for each subgroup. The analysis will be conducted independently in each country.

Whilst it is possible to use regression to build a predictive model, our project hews to the principles of positive selectivism that underlie the WHO conceptualisation of ‘health for all’ and Universal Health Coverage. In contrast to negative selctivism (individual-focused means-testing) WHO, the World Bank, the Lancet Global Health Commission on Global Eye Health and others call for health services to be extended on the basis of sociodemographic group membership. As such, we are primarily interested in identifying which subgroups have the lowest adjusted odds of attendance, rather than the predicted probability of attendance for an individual based on their characteristics.

Whilst our analysis will involve multiple comparisons, we will not use Bonferroni or other adjustments because our primary aim is identifying the 2–3 sociodemographic subdomains associated with the lowest attendance rates. We note that false positives are not associated with clinical risk in this project.

### Analytic plan

We will use the following steps to perform the statistical analysis:

1. Count the number of people in each subgroup (e.g. males, females).
2. Calculate the proportion of people who attended within each subgroup.
3. Use univariable logistic regression with to assess the simple association between each subdomain and attendance, one at a time.
  a. We will present the odds ratio for each association with its p value and a 95% confidence interval.
  b. Associations where p<0.05 will be used to fit a multivariable model.
4. We will compare crude and fully adjusted estimates to assess whether there is any likely confounding.
5. We will then consider whether any of the variables lie on the causal pathway between any others, aided by the development of a causal loop diagram.
6. We will use pairwise testing to check for effect modification, examining whether the effect of any variable differs by the category of another variable. This will help us examine intersectionality.
7. We will plot a correlation matrix and use the Variance Inflation Factor for each variable to assess collinearity.
8. Based on these findings, we will fit a final, fully adjusted model that includes all independent variables. We will use this model to estimate the adjusted odds of attendance for each subdomain along with p values and 95% confidence intervals.
9. These values will be presented in a summary table and used to generate a coefficient plot.

We will summarise the output using the template table below ( Table 11).

**Table 11.** Template table for summarising the regression output.

Characteristic	Category	n (%)	Attended n (%)	Univariate			Multivariate		
				Odds ratio	p	95%CI	Odds ratio	p	95%CI
Gender	Female								
	Male								
Age	N/A								
Location	Urban								
	Rural								
Ethnicity	Dominant ethnicity (e.g. Batswana)								
	Ethnicity 2								
	Ethnicity 3								
	Ethnicity n...[list all on individual lines]								
Language	Dominant language (e.g. Setswana)								
	Language 2								
	Language n...[list all on individual lines]								
Religion	Dominant religion								
	Religion 2								
	Religion n [list all on individual lines]								
Health problems	Yes								
	No								
Household size	1								
	2								
	...n [list all up to 15+ on individual lines]								
Concrete floor	Yes								
	No								
Social welfare receipt	Yes								
	No								
Parents' job	Professional/office								
	Skilled manual								
	Unskilled manual								
	Domestic								
Parents' nationality	Batswana								
	Other								
Household members	Mother								
	Father								
	Grandmother								
	Grandfather								
	Aunt								
	Uncle								
	Siblings								
	Others								

Based on our output, it will be possible to rank all of the subgroups according to adjusted odds of attendance. As noted above, our overall aim is to identify the sociodemographic groups with the lowest overall attendance, so that we can then work with representatives from these groups to identify barriers and potential solutions. In the spirit of proportionate universalism, we are hoping that solutions suggested by these groups will improve the overall mean attendance rate whilst delivering the greatest benefit to groups with the greatest baseline need.

### Process of selecting the lowest group(s)

We want to ensure that our selected ‘left behind’ subgroup represents at least 10% of the total study population. This is because our follow-on work will develop and test interventions to try and improve attendance and we need to have a large enough pool of non-attenders to work with and then test the interventions.

There is a good chance that one single sociodemographic subgroup that contains >10% of the total population will be found to have the lowest adjusted odds of attendance with a p value of <0.05. However, it is also possible that a tiny group (i.e. reflecting a rare characteristic) is found to have the lowest overall odds. If this is the case, we will include the group(s) with the next-lowest overall odds until the included groups collectively represent >10% of the total study population.

### Bias

To reduce the risk of selection bias, the sociodemographic questions will be asked of all those referred to triage clinics. We have developed robust sets of sociodemographic questions that minimise the risk of recall bias, and we will deliver standardised training to reduce the risk of measurement bias.

Referred participants will be given an appointment date at a local clinic for a free follow-up provided by the local eye service. In the vast majority of the places where screening programmes operate there simply are not any alternative providers. In cases where there are other providers, they are unlikely to be free. Nevertheless, we cannot rule out instances where non-attenders have not faced barriers to accessing care, they have simply sought care from other qualified providers. If some groups are more likely than others to seek care elsewhere this will bias our findings. Subsequent phases of our research will involve interviewing non-attenders to explore why they were not able to access our clinics, and at this stage we will be able to assess the extent to which this is a problem. For our current study we will assume that all non-attenders have not managed to access adequate care.

### Data management

Any participants’ identifiable data collected by the Study Coordination Centre will be stored securely and their confidentiality protected in accordance with the Data Protection Act 1998 on Peek Vision servers.

Data and all appropriate documentation will be stored for a minimum of 12 months after the completion of the study, including the follow-up period.

All analyses will be performed on anonymised data (name, date of birth, and address removed), held on encrypted and password-protected servers at LSHTM.

Data will be collected by eye care programme providers using Android devices with access to the Peek Capture application. Peek Capture enforces security controls that include strong device passcodes and native Android encryption. Data stored is time limited, the device syncs via an encrypted connection with a Peek managed server, the data is then deleted to minimise the risk of data stored on the device.

The data will be stored on a Peek managed server hosted in a Virtual Private Cloud (VPC) utilising the Amazon Web Services (AWS) Cloud. Each Peek powered programme will be hosted on its own dedicated server and a VPC that will reside in the UK/EU ensuring all of the data privacy safeguards as governed under the GDPR. All data collected will be securely stored in AWS data centers which are state of the art, utilising innovative architectural and engineering approaches.

### Ethical considerations

***Ethical review***

We have already obtained ethical approval from the LSHTM ethics committee, and ethics committees in Botswana, India, Kenya and Nepal.

***Risks and Benefits***

There are no direct benefits to participants. The information gleaned from the study will help us to identify and engage with the groups that are least likely to attend in attempt to improve access to those groups.

There are two main risks. Firstly, some participants may experience psychological discomfort when asked about their life circumstances, particularly if they are very disadvantaged or ashamed of their social and/or material conditions. Members of persecuted or marginalised ethnic, religious, or social groups may be afraid to disclose this information. The second risk is inadvertent disclosure of sensitive and confidential personal information.

In terms of mitigating these risks, we have developed sociodemographic questions with in-country teams and lay review in order to minimise the risk of causing distress. We will pilot the questions and revise the wording further if any issues arise. Sociodemographic questions will be asked in a confidential setting where others will not be able to hear the responses. Programme implementers will be trained to protect privacy and confidentiality during data collection. Programme implementers will also receive training on how to support participants who become distressed. This includes giving them time and space, offering supportive comments, and providing contact details for local support groups.

We are using world-class data management and storage processes to provide the highest possible level of protection for patient data. See the data management section below.

***Consent***

No participants will be placed under any compulsion or coercion. Participants will not have to provide consent in order to participate in- and benefit from the screening programmes. Participants will be able to decline to answer as many questions as they wish. We will use tick boxes rather than signatures to obtain written consent among non-literate groups. We will read out the study information and consent form for non-literate participants and provide impartial witnesses who will also sign the consent form in these instances.

***Adult consenting procedure (in-person)***

All adult participants will be asked to provide informed written (digital tick box) consent for their anonymised data to be published at the point that they present to services. Their consent will be taken by data collectors, and their consent status will be recorded in the Peek Acuity app.

For this negligible-risk study, the patient information leaflet (PIL) will be read to each adult:

“Now I will ask you a series of questions about your income, education, occupation, and personal characteristics.
We will use this information to check that our programme is reaching people from every background; and to make improvements where we are missing certain groups.
We will anonymise your data and keep it safe and secure on a virtual server within the European Union (EU).
We will not sell your data.
We will publish our findings in a research journal and a public repository, but your personal information will not be included.
You do not have to let us analyse and share your data; participation is completely voluntary. You can change your mind at any time and your decision will not affect the care you receive
We have a researcher available to answer any questions you may have [may be via phone]
Please read this statement and tick to indicate whether you consent or not:”

Potential participants will have the option to read the information for themselves if they wish. Each person will be asked if they would like to ask any questions or discuss the study further. If not, they will be handed the android device displaying the following text and tick boxes:

*“I understand that my anonymous data may be shared with other researchers or online in a public repository, and that I will not be identifiable from this information. I understand that my decision will not affect the care that I receive, and I am free to change my mind anytime I like.”*
[ ] I consent
[ ] I do not consent

Implementers will be given training on taking consent and dealing with questions as part of their orientation training.

The use of a tick box rather than a signature aligns with the MHRA/HRA joint guidance for low-risk trials ^30^ and extends participation to those who may not be able to write their names.

The statement will be available in all the major local languages. The relevant domestic technical working group for each country will perform the translation. The translation will be checked by a lay representative.

A consent statement will be read out to those who cannot read - this includes people with marked visual impairment. These people will be given the opportunity to provide verbal consent and tick digital boxes on the Android device to signal their consent if they agree to participate. Independent witnesses will be available at each screening site to co-sign the consent form for all illiterate participants. These witnesses will be provided by primary care centre for community screening programmes. There will not be any house-to-house screening.

**Table:** Digital form used to obtain consent from adults who cannot read.

Statement to be read out by programme implementer	Participant’s tick	Impartial witness tick
“I have had the information explained to by study personnel in a language that I understand. I have had the opportunity to consider the information, ask questions and have these answered satisfactorily.”		
“I understand that my participation is voluntary and that I am free to withdraw at any time without giving any reason, without my medical care or legal rights being affected.”		
“I understand that data about me may be shared via a public data repository or by sharing directly with other researchers, and that I will not be identifiable from this information.”		
“I agree to take part in the above named study.”		

### Obtaining remote consent for children

As parents/guardians will not be present at school on the day that screening teams attend, we will send participant information and consent forms in the week before the screening teams arrive. Depending on the programme, this will either be by SMS or a paper form sent home with the children. The material will be written in the local dialect and will provide a free-phone number, email address, and postal address to discuss any questions with the study with the in-country research manager.

We have worked with ethics committees at LSHTM and in the respective study site countries to develop a consent process that is proportionate to the level of risk. All ethics committees have approved written opt-out consent, given the low-risk nature of the project. All parents will be sent an SMS or paper form from the school and asked to reply/return a signed form only if they do not want their child to participate. Illiteracy is an issue, however schools routinely send material home with children for their parents and in each instance, we have been reassured by school representatives that simple processes are in place to support illiterate parents/guardians, such as having a literate friend or family member read the material to them, or having the teacher personally relay the information.

SMS consenting messages:

1. Hi! When we check your child's vision next week, we will also ask them a series of questions about their home situation and personal characteristics
2. We will use this information to check that our programme is reaching people from every background; and to make improvements where we are missing certain groups
3. We will anonymise all data and keep it safe and secure on a virtual server within the European Union (EU) for 10 years // We will not sell your child's data
4. We will publish our findings in a research journal and a public repository, but your child's personal information will not be included
5. There are no direct benefits or risks to you or your child. // The [Name] ethics board has approved this project.
6. Participation is voluntary. You can change your mind at any time // You can find more information here [bit.ly hyperlink] // Or free-phone +xxxxxxxxxxxxxxx
7. Please read the next two messages very carefully. They set out a consent statement. Once you have read them, please respond if you DO NOT agree
8. I understand that my child's anonymised data may be shared with other researchers or online in a public repository for research
9. I understand that I can call +xxxxxxxxxxxxxxx to ask any questions; my decision will not affect the care my child receives; and I can change my mind at any time
10. If you DO NOT consent for us to use your child's data, please reply to this message with your full name // Your message will be free

In areas where teams are not able to use SMS data collection systems, we will use paper forms sent home with children for their parents to sign. The forms will contain the same information, along with a tick box to provide consent.

If the parents/guardians are illiterate, they will be provided with a phone number to speak with a research coordinator. If they are unable to use the provided phone number, parents/guardians will be asked by the teachers to attend with their child on the day of screening to provide verbal consent.

### Child assent

Assent will be sought from children by programme implementers before asking the sociodemographic questions:

“Now I am going to ask you some questions about you and your home life. You can say ‘I don’t know’ or skip any questions that you don’t want to answer. Please tick this [digital] box to show that you understand what I’ve just said.”

***Procedures for following-up non-attenders***

All participants who do not present for treatment within locally set timeframes (generally 3–4 weeks from the date of referral) will receive SMS reminders and the in-country programme team will have access through Peek Admin to contact non-attenders by SMS or telephoning all non-attending patients.

## Dissemination

Our findings will be shared with programme managers who will use the information to target sociodemographic groups with the lowest attendance rates. Managers will engage with members of these left-behind groups to explore the specific barriers they are facing and develop potential service improvements that will be trialled. Our findings will also be shared with screening partners around the world who are not currently analysing outcomes by sociodemographic indicators. We will publish our findings in a peer-reviewed journal, present the findings at conferences, and develop policy briefs to share with governments in each country.

### Protocol study status

This was true at the time of submission. Now we have approval from ethics boards in the UK, Botswana, India, Kenya and Nepal. Recruitment has started in all four countries.

## Data Availability

OSF: Data Management Plan https://osf.io/dyj3f/ (DOI: 10.17605/OSF.IO/DYJ3F) ^31^ This project contains the following underlying data: Appendix_DMP.docx Data are available under the terms of the Creative Commons Zero "No rights reserved" data waiver (CC0 1.0 Public domain dedication).
