# Supplementary material for: Sociodemographic characteristics of community eye screening participants: protocol for cross-sectional equity analyses in Botswana, India, Kenya, and Nepal
Source: Wellcome Open Res. 2023 Nov 16;7:144. Originally published 2022 May 4. [Version 2] doi: 10.12688/wellcomeopenres.17768.2 (PMC10357071; doi:10.12688/wellcomeopenres.17768.2)
Supplement: Peer review history

# Reviewer report: Reviewer response for version 2

**DOI:** 10.21956/wellcomeopenres.22377.r70133

**Published:** 2025-06-13

**Authors:** Srinivas Marmamula

**Affiliations:** 1 Brien Holden Institute of Optometry and Vision Science, GPR ICARE, L V Prasad Eye Institute, Hyderabad, Telangana, India

All my comments are adequately addressed. I recommend acceptance.

Is the study design appropriate for the research question?

Yes

Is the rationale for, and objectives of, the study clearly described?

Yes

Are sufficient details of the methods provided to allow replication by others?

Yes

Are the datasets clearly presented in a useable and accessible format?

Yes

Reviewer Expertise:

Public Eye Health; Community Eye Care; Epidemiology; Primary Eye Care

I confirm that I have read this submission and believe that I have an appropriate level of expertise to confirm that it is of an acceptable scientific standard.

# Reviewer report: Reviewer response for version 2

**DOI:** 10.21956/wellcomeopenres.22377.r70131

**Published:** 2023-12-08

**Authors:** Isabelle Jalbert

**Affiliations:** 1 The University of New South Wales, Kensington, Australia

I have reviewed the responses and revisions submitted by the authors and confirm that these are appropriate. I am happy for the status to be amended to Approved for this article.

Is the study design appropriate for the research question?

Yes

Is the rationale for, and objectives of, the study clearly described?

Yes

Are sufficient details of the methods provided to allow replication by others?

Yes

Are the datasets clearly presented in a useable and accessible format?

Partly

Reviewer Expertise:

NA

I confirm that I have read this submission and believe that I have an appropriate level of expertise to confirm that it is of an acceptable scientific standard.

# Reviewer report: Reviewer response for version 2

**DOI:** 10.21956/wellcomeopenres.22377.r70132

**Published:** 2023-11-16

**Authors:** Afua O. Asare

**Affiliations:** 1 The University of Utah, Salt Lake City, Utah, USA

Prior comments and suggestions have been sufficiently addressed and considered.

Is the study design appropriate for the research question?

Yes

Is the rationale for, and objectives of, the study clearly described?

Yes

Are sufficient details of the methods provided to allow replication by others?

Partly

Are the datasets clearly presented in a useable and accessible format?

Not applicable

Reviewer Expertise:

Health services research, public health, pediatric vision screening

I confirm that I have read this submission and believe that I have an appropriate level of expertise to confirm that it is of an acceptable scientific standard.

# Reviewer report: Reviewer response for version 1

**DOI:** 10.21956/wellcomeopenres.19665.r59693

**Published:** 2023-07-19

**Authors:** Afua O. Asare

**Affiliations:** 1 The University of Utah, Salt Lake City, Utah, USA

**Strengths**

- Stakeholder-input ensures relevance for each country context

**Weaknesses**

- There appears to be assumptions that readers have prior familiarity with Peek Vision programs and activities because of lack of explanation of some processes and the use of certain jargon e.g., GitHub software. Please see detailed comments below for more information.
- Some of the decisions regarding the study design appear arbitrary. Further details of decisions regarding the study design are needed.
- Some concerns about soliciting the true consent and approval of parents.

**Detailed Comments**

Introduction

*PG 3, paragraph 6 -* Where and when is triage offered? Is it same day at the same location or not? Please clarify.

Methods

*PG 4, Study design *– replace ‘two points’ with ’two time points' (or something similar) so that readers are clear that you are referring to durations in time.

*PG 4, Study design *–What steps is the research team taking to ensure a representative sample?

*PG 5, Setting, paragraph 1 *- Why 6 and 12 months? Explain the rationale. Are these the two points in the screening program that were previously mentioned in the study design section? Please make it clear if it is.

*PG 5, Setting, paragraph 1 *- The choice of 1,000 participants appears arbitrary. How was this number chosen?

*PG 5, Closure, paragraph 4* – It is unclear why only 100 participants will be chosen for the Nepal study? Also, please clarify how researchers will select the 1,000 participants. Will they select an equal number of participants across the countries, consecutive patients (regardless of the context) until 1,000 sample is reached? Please clarify. Similarly, please clarify the choice of 365 days.

*PG 7, Table 3 (Phone ownership adult response options)* - I assume the question posed in the protocol is different from what was actually proposed in the survey? If so, consider including a note at the end of the table indicating so. In its current state, it is unclear what these multiple-choice options represent. Are they the persons that receive texts on behalf of the patient? Personally, I would suggest that these choices be written as follows (or similar format):

 - Yes, my mother or father

 - Yes, my spouse

 - Yes, my daughter or son

 etc.

*PG 7, Table 3 (Child response options)* – Similar comment as above.

*PG 7, Table 3 (Distance from screening location to clinic, Notes) *– Unclear what clinic they are referring to. Clinic for follow-up? Clinic for triage or for treatment? Please clarify. Also, what happens if there is more than one clinic in the area?

*PG 7, Table 3 (Education, Child response options) *– Are the authors assuming all children within the target age group will be enrolled in schools? What is this assumption based on? Is screening only offered in schools? If so, consider including a note at the bottom of the table to clarify for readers (even if this has been mentioned earlier).

*PG 26, Outcome measures, 1 ^st^ paragraph *– Please explain the reasoning behind choosing 21 days as the cutoff.

*PG 26, paragraph 3, Statistical methods* – It is unclear what GitHub is. Please describe this tool briefly.

*PG 28, Adult consenting procedure (in-person) last but one bullet point–* The first sentence in unclear. Please clarify/re-write. Should it read something like this? “We will not analyze and share your data without your prior permission”.

*PG 29, Obtaining remote consent for children*- How will they ensure that the true consent of parents has been given? And that parents truly understand their rights? Also, there is no description on how the paper consent forms will be collected or returned to the research team. Please clarify if it is an ‘opt out only’ process. In other words, are parent only required to return a signed consent form if they wish to opt out? Or are you requesting all paper forms (and SMS) be completed and returned regardless of parent’s decision. In low resource/SES communities’ participation rate may be low if consent is required of all. Conversely, some children may inadvertently participate in the study though parents had not intended for them to. Finally, how will illiterate individuals go through the consent process? They may be unable to read the languages written.  

**Typos:**

1. *2 ^nd^ paragraph, 2 ^nd^ sentence.* “Eye conditions exhibit marker inter- and intra-national inequalities in disease rates, access to care, and outcomes, with poorer, rural women often facing the highest barriers to accessing care7–9.” **marked **instead of **marker**
2. *Last paragraph on page 2* “Those whose vision does not meet pre-set threshold ‘screen positive’ and are sent to triage (which may be co-located with the screening operation, or may be performed at another”. Please omit ‘are’

Is the study design appropriate for the research question?

Yes

Is the rationale for, and objectives of, the study clearly described?

Yes

Are sufficient details of the methods provided to allow replication by others?

Partly

Are the datasets clearly presented in a useable and accessible format?

Not applicable

Reviewer Expertise:

Health services research, public health, pediatric vision screening

I confirm that I have read this submission and believe that I have an appropriate level of expertise to confirm that it is of an acceptable scientific standard.

# Response

**Published:** 2023-10-17

**Authors:** luke allen

**Affiliations:** London School of Hygiene & Tropical Medicine, UK

**Reviewer 3**

***Strengths***

 Stakeholder-input ensures relevance for each country context  

***Weaknesses***

 There appears to be assumptions that readers have prior familiarity with Peek Vision programs and activities because of lack of explanation of some processes and the use of certain jargon e.g., GitHub software. Please see detailed comments below for more information.  

 Some of the decisions regarding the study design appear arbitrary. Further details of decisions regarding the study design are needed.  

 Some concerns about soliciting the true consent and approval of parents.  

***Detailed Comments***  

 Introduction  

 PG 3, paragraph 6 - Where and when is triage offered? Is it same day at the same location or not? Please clarify.  

- The setup and flow of Peek programmes varies according to context. We have added further text on p2 to emphasise this. At the first mention of triage we already state that triage “may be co-located with the screening operation, or may be performed at another time/place”. We have added the following text directly after this sentence: *For example, in some school-based programmes, triage happens in a room next door immediately after visual acuity screening. Whereas in some door-to-door community-based programmes, those who screen positive are referred on to attend the local primary care facility a week or so later.*    

Methods  

 PG 4, Study design – replace ‘two points’ with ’two time points' (or something similar) so that readers are clear that you are referring to durations in time.  

- Corrected

PG 4, Study design –What steps is the research team taking to ensure a representative sample?  

- This is covered lower down in the text, however we have amended the wording on p4, replacing ‘representative sample’ with ‘from every 10 ^th^ person who presents to screening’.

PG 5, Setting, paragraph 1 - Why 6 and 12 months? Explain the rationale. Are these the two points in the screening program that were previously mentioned in the study design section? Please make it clear if it is.  

- These timepoints were initially mooted by programme leads to align with routine multistakeholder review meetings. We have removed the wording as it is not germane to our study or the analyses that we will perform.  

PG 5, Setting, paragraph 1 - The choice of 1,000 participants appears arbitrary. How was this number chosen?  

- This has been removed. We will use a sample size of 3,850 in each country. An updated methods section explains how we arrived at this number: We are aiming to compare odds of attendance between sociodemographic subgroups. The number of people in each subgroup is the factor that will determine our ability to make statistically significant comparisons for a given level of α, as well as determining the probability of type II error (i.e. incorrectly rejecting the null hypothesis). With a 95% confidence level, a 5% margin of error, and a maximally conservative proportion of 0.5, we would need to have at least 385 people in each subgroup to make statistically significant comparisons between groups.   In each study site we will collect data from the first 3,850 consecutive consenting people who are referred. This will enable us to make statistically significant comparisons between groups that contain at least 10% of the overall population. 

PG 5, Closure, paragraph 4 – It is unclear why only 100 participants will be chosen for the Nepal study? Also, please clarify how researchers will select the 1,000 participants. Will they select an equal number of participants across the countries, consecutive patients (regardless of the context) until 1,000 sample is reached? Please clarify. Similarly, please clarify the choice of 365 days.  

- We have fully revised this section: In each study site we will collect data from the first 3,850 consecutive consenting people who are referred. This will enable us to make statistically significant comparisons between groups that contain at least 10% of the overall population. Data will be collected by screening staff/volunteers working in each setting as part of the routine screening process that is conducted using the Peek Vision app.  

PG 7, Table 3 (Phone ownership adult response options) - I assume the question posed in the protocol is different from what was actually proposed in the survey? If so, consider including a note at the end of the table indicating so. In its current state, it is unclear what these multiple-choice options represent. Are they the persons that receive texts on behalf of the patient? Personally, I would suggest that these choices be written as follows (or similar format):   - Yes, my mother or father   - Yes, my spouse   - Yes, my daughter or son   etc.  

 PG 7, Table 3 (Child response options) – Similar comment as above.  

- We have updated the wording as suggested for adults and children.

PG 7, Table 3 (Distance from screening location to clinic, Notes) – Unclear what clinic they are referring to. Clinic for follow-up? Clinic for triage or for treatment? Please clarify. Also, what happens if there is more than one clinic in the area?  

- Table text updated to clarify ‘triage clinic’. We also now note that referred participants are given an appointment at a specific clinic (there often are no other options anyway).

 PG 7, Table 3 (Education, Child response options) – Are the authors assuming all children within the target age group will be enrolled in schools? What is this assumption based on? Is screening only offered in schools? If so, consider including a note at the bottom of the table to clarify for readers (even if this has been mentioned earlier).  

- Clarification added to the ‘notes’ column. All national child eye screening programmes in our study sites are implemented as school-based programmes.

PG 26, Outcome measures, 1st paragraph – Please explain the reasoning behind choosing 21 days as the cutoff.  

- We have added the following text: *This cut-off has been selected because most appointments are made for one week after triage, and very few people attend more than two-weeks after their appointed date.*  

PG 26, paragraph 3, Statistical methods – It is unclear what GitHub is. Please describe this tool briefly.   

- GitHub is a free online repository where researchers upload open access data and code. However, we are not tied to this particular website. As such we have deleted this text and replaced it with the following: We will publish anonymised aggregate data online, along with all our statistical code.      

PG 28, Adult consenting procedure (in-person) last but one bullet point– The first sentence in unclear. Please clarify/re-write. Should it read something like this? “We will not analyze and share your data without your prior permission”.  

- A couple of words were missing from the sentence, thank you for spotting this. The sentence now reads: You do not have to let us analyse and share your data; participation is completely voluntary.  

PG 29, Obtaining remote consent for children- How will they ensure that the true consent of parents has been given? And that parents truly understand their rights? Also, there is no description on how the paper consent forms will be collected or returned to the research team. Please clarify if it is an ‘opt out only’ process. In other words, are parent only required to return a signed consent form if they wish to opt out? Or are you requesting all paper forms (and SMS) be completed and returned regardless of parent’s decision. In low resource/SES communities’ participation rate may be low if consent is required of all. Conversely, some children may inadvertently participate in the study though parents had not intended for them to. Finally, how will illiterate individuals go through the consent process? They may be unable to read the languages written.   

- The following text has been added: We have worked with ethics committees at LSHTM and in the respective study site countries to develop a consent process that is proportionate to the level of risk. All ethics committees have approved written opt-out consent, given the low-risk nature of the project. All parents will be sent a paper form from the school and asked to return a signed form only if they do not want their child to participate. Illiteracy is an issue, however schools routinely send material home with children for their parents and in each instance we have been reassured by school representatives that simple processes are in place to support illiterate parents/guardians, such as having a literate friend or family member read the material to them, or having the teacher personally relay the information.  

Typos: 2nd paragraph, 2nd sentence. “Eye conditions exhibit marker inter- and intra-national inequalities in disease rates, access to care, and outcomes, with poorer, rural women often facing the highest barriers to accessing care7–9.” marked instead of marker  

- Corrected.

  Last paragraph on page 2 “Those whose vision does not meet pre-set threshold ‘screen positive’ and are sent to triage (which may be co-located with the screening operation, or may be performed at another”. Please omit ‘are’  

- Sentence re-worded.

# Reviewer report: Reviewer response for version 1

**DOI:** 10.21956/wellcomeopenres.19665.r56944

**Published:** 2023-07-18

**Authors:** Isabelle Jalbert

**Affiliations:** 1 The University of New South Wales, Kensington, Australia

This well written manuscript presents a study protocol for quantifying the associations between sociodemographic characteristics and vision screening attendance in low- and mid-income countries. The study aim, rationale, research question and hypothesis including the key problem (e.g., *approximately half of those "screen positive" do not attend for further assessment and treatment, and these are more likely to be women and girls*) are clearly presented.

 Methods:

1. Is there a discrepancy between the text, Table 1 and Figure 1? The manuscript text and Table 1 appear to suggest that Peek-programmes scaling up in 2022 will be located in Botswana, Kenya, and Nepal whereas the Figure legend in Figure 1 appears to suggest this would be Botswana, Kenya, and Pakistan. Can you confirm that this is correct?
2. Survey development (items 4 and 5). The methods are well described but was co-design/participatory research design with involvement of participants from the individual countries in the multistakeholder workshops considered and if not, why not?
3. Review inconsistencies in the use of appropriate tense throughout the manuscript. For example in 6. Translation and piloting, the manuscript details how the finalised survey instrument "will be translated" but in the next sentence there is mention of how refinements to optimise the response options "were incorporated and re-translated". 
4. Analysis. One presumes that the data analysis will be stratified by country - this could be more clearly outlined in the description.
5. Ethical consideration. Provide confirmation that ethics approval will be obtained for the "OPT OUT" method for obtaining remote consent for children prior to proceeding with data collection as it is possible that some IRB may have concerns with this approach.

Minor additional comments:

 Page 5, abbreviation SES is not defined. Should you explain whether or how this differs from SD?

 Figure 2: Provide an Y-axis scale for this Figure including units of measurements or specify if the bars are not to be taken as "to scale".

Is the study design appropriate for the research question?

Yes

Is the rationale for, and objectives of, the study clearly described?

Yes

Are sufficient details of the methods provided to allow replication by others?

Yes

Are the datasets clearly presented in a useable and accessible format?

Partly

Reviewer Expertise:

Health Systems Research

I confirm that I have read this submission and believe that I have an appropriate level of expertise to confirm that it is of an acceptable scientific standard, however I have significant reservations, as outlined above.

# Response

**Published:** 2023-10-17

**Authors:** luke allen

**Affiliations:** London School of Hygiene & Tropical Medicine, UK

**Reviewer 2**

 This well written manuscript presents a study protocol for quantifying the associations between sociodemographic characteristics and vision screening attendance in low- and mid-income countries. The study aim, rationale, research question and hypothesis including the key problem (e.g., approximately half of those "screen positive" do not attend for further assessment and treatment, and these are more likely to be women and girls) are clearly presented.  

 Methods: Is there a discrepancy between the text, Table 1 and Figure 1? The manuscript text and Table 1 appear to suggest that Peek-programmes scaling up in 2022 will be located in Botswana, Kenya, and Nepal whereas the Figure legend in Figure 1 appears to suggest this would be Botswana, Kenya, and Pakistan. Can you confirm that this is correct?

- The (outdated) figure has been removed.

Survey development (items 4 and 5). The methods are well described but was co-design/participatory research design with involvement of participants from the individual countries in the multistakeholder workshops considered and if not, why not?  

- Despite calling for lay representation in the early process of identifying sociodemographic domains, none of the four country teams were able to identify and recruit an appropriate representative for this task in time. All countries have now set up formal community advisory boards who have reviewed and commented on the final set of questions that are being used in each location. They will also be heavily involved in upcoming validation exercises. This information will be added to the writeup of the findings from these sites.

Review inconsistencies in the use of appropriate tense throughout the manuscript. For example in 6. Translation and piloting, the manuscript details how the finalised survey instrument "will be translated" but in the next sentence there is mention of how refinements to optimise the response options "were incorporated and re-translated".  

- This issue stems from the fact that all sites proceeded at different speeds. The tenses have been amended so that up to point 5 is in past tense and from point 6 onward is in future tense.

Analysis. One presumes that the data analysis will be stratified by country - this could be more clearly outlined in the description.  

- Correct, this has been added.

 Ethical consideration. Provide confirmation that ethics approval will be obtained for the "OPT OUT" method for obtaining remote consent for children prior to proceeding with data collection as it is possible that some IRB may have concerns with this approach.  

- The following clarification has been added:

*Ethics approval has already been sought and granted to use opt-out consent in each country by national ethics committees, as well as the LSHTM ethics committee.*    

 Minor additional comments:  

 Page 5, abbreviation SES is not defined. Should you explain whether or how this differs from SD?  

- ‘SES’ replaced with ‘sociodemographic’ throughout.

  Figure 2: Provide an Y-axis scale for this Figure including units of measurements or specify if the bars are not to be taken as "to scale".  

- Note added that bars are not to be taken as “to scale”.

# Reviewer report: Reviewer response for version 1

**DOI:** 10.21956/wellcomeopenres.19665.r52282

**Published:** 2022-11-17

**Authors:** Srinivas Marmamula

**Affiliations:** 1 Brien Holden Institute of Optometry and Vision Science, GPR ICARE, L V Prasad Eye Institute, Hyderabad, Telangana, India

The authors aim to quantify the association between socio-demographic variables and attendance rates in vision screening programmes. The authors plan to develop a set of socio-demographic questions to be used in community screening programmes based on a literature review. The study is planned in Botswana, Kenya and Nepal.

 Based on the literature review, the authors have identified 11 broad domains that are feasible to be included in the routine data collection system. The authors modified the selected questions in the target countries based on in-person consultations. Data analysis is planned after the first three months or after receiving 1000 responses. The validity and reliability of the questionnaire will be also assessed. The authors present a sound research study design and detailed methodology to achieve the set objectives.

 I have a couple of comments on the manuscript:

- Please mention how you deal with the cases where they visit other service providers not included in the study as referral centres.
- Any specific reason for selecting these three locations?

Is the study design appropriate for the research question?

Yes

Is the rationale for, and objectives of, the study clearly described?

Yes

Are sufficient details of the methods provided to allow replication by others?

Yes

Are the datasets clearly presented in a useable and accessible format?

Yes

Reviewer Expertise:

Public Eye Health; Community Eye Care; Epidemiology; Primary Eye Care

I confirm that I have read this submission and believe that I have an appropriate level of expertise to confirm that it is of an acceptable scientific standard.

# Response

**Published:** 2023-10-17

**Authors:** luke allen

**Affiliations:** London School of Hygiene & Tropical Medicine, UK

**Reviewer 1**

 The authors aim to quantify the association between socio-demographic variables and attendance rates in vision screening programmes. The authors plan to develop a set of socio-demographic questions to be used in community screening programmes based on a literature review. The study is planned in Botswana, Kenya and Nepal.  

 Based on the literature review, the authors have identified 11 broad domains that are feasible to be included in the routine data collection system. The authors modified the selected questions in the target countries based on in-person consultations. Data analysis is planned after the first three months or after receiving 1000 responses. The validity and reliability of the questionnaire will be also assessed. The authors present a sound research study design and detailed methodology to achieve the set objectives.  

 I have a couple of comments on the manuscript: Please mention how you deal with the cases where they visit other service providers not included in the study as referral centres.  

- We have added the following text:

 
*Screening participants are given an appointment date at a local clinic for a free follow-up provided by the local eye service. In the vast majority of the places where screening programmes operate there simply are not any alternative providers. In cases where there are other providers, they are unlikely to be free. Nevertheless, we cannot rule out instances where non-attenders have not faced barriers to accessing care, they have simply sought care from other qualified providers. If some groups are more likely than others to seek care elsewhere this will bias our findings. Subsequent phases of our research will involve interviewing non-attenders to explore why they were not able to attend our clinics, and at this stage we will be able to assess the extent to which this is a problem. For our current study we will assume that all non-attenders have not managed to access adequate care, effectively performing a type of intention-to-treat analysis.*  

 Any specific reason for selecting these three locations?  

- Please note that since submitting the protocol last year we have also had Indian colleagues join the project with a screening programme in Uttar Pradesh. We have added the following text:

 
*These four countries were chosen for the current project because they all have active Peek programmes led by institutions with active research interest and existing relationships with LSHTM.*
